# P-2075. Immunogenicity of co-administration of influenza vaccine and COVID-19 vaccine in healthy volunteers

**DOI:** 10.1093/ofid/ofae631.2231

**Published:** 2025-01-29

**Authors:** Nonthanat Tongsengkee, Nawamin Pinpathomrat, Pisud Siripaitoon, Narongdet Kositpantawong, Siripen Kanchanasuwan, Sorawit Chittrakarn, Sarunyou Chusri

**Affiliations:** Faculty of Medicine, Prince of Songkla University, Hatyai, Songkhla, Thailand; Faculty of Medicine, Prince of Songkla University, Hatyai, Songkhla, Thailand; Faculty of Medicine, Prince of Songkla University, Hatyai, Songkhla, Thailand; Faculty of Medicine, Prince of Songkla University, Hatyai, Songkhla, Thailand; Faculty of Medicine, Prince of Songkla University, Hatyai, Songkhla, Thailand; Faculty of Medicine, Prince of Songkla University, Hatyai, Songkhla, Thailand; Faculty of Medicine, Prince of Songkla University, Hatyai, Songkhla, Thailand

## Abstract

**Background:**

The emergence of coronavirus disease 2019 (COVID-19) and seasonal influenza results in a substantial disease burden worldwide. The immunogenicity of co-administering the COVID-19 and influenza vaccines was not well established, as co-administration may reduce the antibody response. The aim of the study is to evaluate the humoral immune response (HIR) and cell-mediated immune response (CMIR) of co-administration among healthy volunteers.

**Figure:**
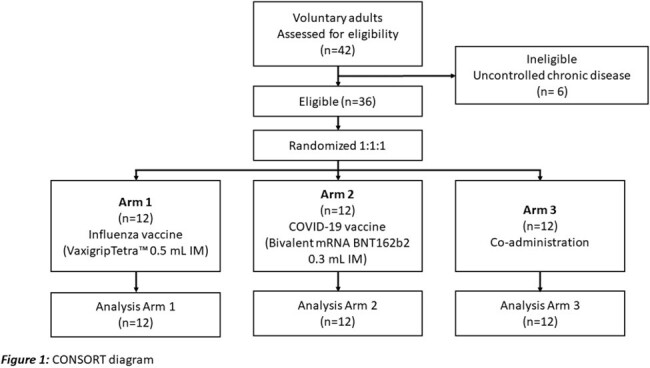

**Methods:**

A pilot randomized controlled trial was conducted in Southern Thailand from March to April 2024. Healthy adults who had received at least one dose of any COVID-19 vaccine more than 6 months prior were enrolled and randomly assigned (1:1:1) to receive the influenza vaccine (VaxigripTetra™) alone, the COVID-19 vaccine (bivalent mRNA BNT162b2) alone, or co-administration of both vaccines. T-cell response against SARS-CoV-2 by Interferon gamma enzyme-linked immunosorbent spot (IFN-γ ELISpot) assay and anti-receptor-binding domain immunoglobulin G (anti-RBD IgG) level were measured before and 4 weeks after vaccination. The primary outcomes were the geometric mean ratio (GMR) of ELISpot and anti-RBD IgG level, with a lower bound 95% confidence interval (CI) > 0.67 for the non-inferiority margin (within 1.5-fold). The sample size was 12 per arm, according to the pilot study.

**Figure:**
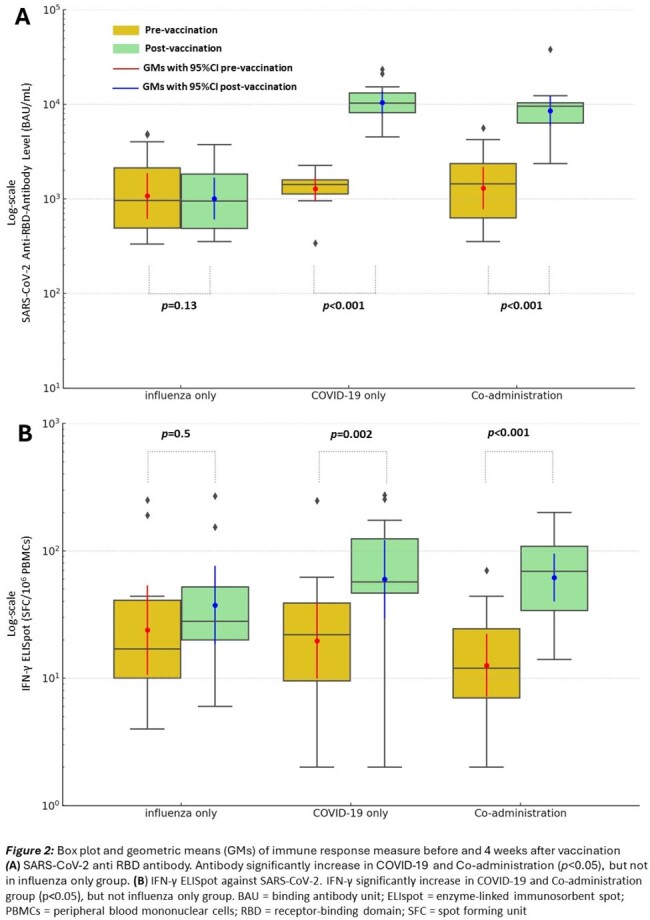

**Results:**

Thirty-six participants were recruited. The enrollment and study flow are shown in Figure 1. Demographic data of participants are demonstrated in Table 1. Geometric means (GMs) for IFN-γ ELISpot and anti-RBD IgG are shown in Figure 2. GMRs between the co-administration and COVID-19 group were 1.03 (95% CI 0.43-2.44) for IFN-γ ELISpot and 0.82 (95% CI 0.52-1.29) for anti-RBD IgG, both failed to meet the non-inferiority margin due to the lower bound 95% CI < 0.67 (Figure 3). Mild solicited systemic adverse reactions in the influenza, COVID-19, and Co-administration groups were 8.3%, 75%, and 83%, respectively.

**Figure:**
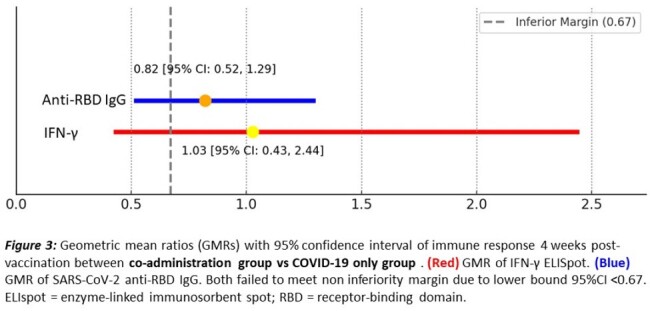

**Conclusion:**

Co-administration of COVID-19 and influenza vaccines tended to show comparable both HIR and CMIR, as well as adverse reactions, with COVID-19 vaccination alone. This finding may support co-administration policy. According to pilot study, larger sample size study and effectiveness of vaccine co-administration need to be investigated.

**Figure:**
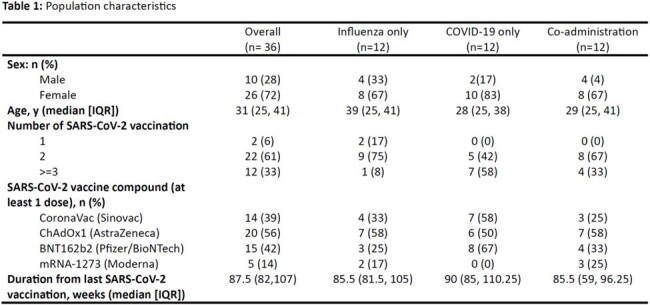

**Disclosures:**

All Authors: No reported disclosures

